# Supplementary material for: PEERS — An Open Science “Platform for the Exchange of Experimental Research Standards” in Biomedicine
Source: Front Behav Neurosci. 2021 Oct 21;15:755812. doi: 10.3389/fnbeh.2021.755812 (PMC8567102; doi:10.3389/fnbeh.2021.755812)
Supplement: Supplementary file 1 [file Data_Sheet_1.pdf]

Supplementary Information

Table S1: Factors with references embedded for the Open Field protocol

Category	Factor	Literature/Reference
**Generic - Design Rigor**	Establishing whether an experiment informs a formal knowledge claim or not	
	Blinding during experiment and during outcome assessment and analysis	10.1186/1744-9081-7-48
	Randomization of allocation and documentation of method used	
	Sample size and rationale behind sample size	
	Criteria for outliers	
	Inclusion and exclusion criteria or data censoring	10.3389/fneur.2020.00650
	Matching or balancing sex of animals/treatment of allocation	10.1186/1744-9081-7-48
	Addressing confounds with treatment/setting/co-morbidities	
**Generic - *in vivo*- Subjects**	Age	https://doi.org/10.2466/pms.1977.45.3f.1059<br>10.1002/npr2.12052
	Body Weight	10.3390/ani6010004<br>10.1258/002367796780744901<br>10.1007/s11011-017-0140-z
	Strain - incl. Substrain	10.1007/s10517-015-2821-0<br>10.1538/expanim.60.111
	Breeder	https://www.frontiersin.org/articles/10.3389/fnins.2015.00424/full<br>https://doi.org/10.1016/j.physbeh.2012.12.019
	Transport from Breeder	
	Standardisation of husbandry practice	10.1111/gbb.12149<br>https://doi.org/10.1016/j.neuroscience.2013.02.012<br>https://doi.org/10.1016/j.physbeh.2017.04.009<br>10.1111/j.1601-183X.2005.00121.x
	Acclimatization to holding facility prior to experimentation	
	Sex	https://doi.org/10.1016/S0031-9384(01)00494-2<br>https://doi.org/10.1002/ajmg.c.31565<br>https://doi.org/10.1016/j.neuroscience.2013.02.012<br>10.1016/j.bbr.2011.03.038
	Estrous cycle	10.1111/j.1467-9450.1994.tb00946.x<br>https://doi.org/10.1016/S0031-9384(01)00494-2<br>https://doi.org/10.1111/j.1601-183X.2006.00249.x
	Gonadal Hormones	10.1016/j.psyneuen.2017.10.007<br>10.1016/j.yhbeh.2008.10.010<br>10.1016/s0006-8993(02)03567-9<br>10.1016/s0306-4522(98)00341-8<br>10.1037/a0012749<br>10.1016/s0003-3472(72)80145-3<br>10.1037/0735-7044.118.2.306<br>10.1016/0031-9384(73)90124-8
	Weaning age	https://doi.org/10.1371/journal.pone.0167652

	Gut Microbiome	10.1038/s41398-018-0240-5
	Specific Pathogen Free	10.1111/nmo.12110<br>10.1016/j.psyneuen.2014.01.014<br>10.1080/01616412.2019.1675021<br>10.1371/journal.pone.0201829
	Description of controls and their suitability for the experiment	10.1073/pnas.0912955107<br>https://doi.org/10.1371/journal.pbio.3000411
	Recording of physiological variables during experiment	10.1186/1744-9081-7-48
**Generic- *in vivo*- Housing**	Home cage type (Size, Color, etc)	10.1016/j.physbeh.2005.01.008<br>10.1016/S0166-2236(00)01718-5<br>10.1016/S0006-8993(98)00735-5
	Home cage bedding type	10.1037/com0000147<br>10.1016/j.physbeh.2013.08.003
	Enrichment	https://doi.org/10.3389/fnbeh.2014.00257
	Room Temperature	10.1016/j.pbb.2006.10.005
	Room Humidity	
	Light intensity/type	10.1111/j.1601-183X.2005.00121.x
	Circadian Rhythm/Light Cycle	10.3791/51785<br>10.18632/aging.100142<br>10.1016/j.bbr.2009.07.001<br>10.1016/j.neures.2007.06.1474<br>10.1034/j.1601-183x.2003.00002.x
	Acoustic noise (also radio)	10.1258/la.2009.0080098<br>PMID: 20587160
	Ultrasound noise	
	Vibration noise	
	Group or isolated housing	10.1111/j.1601-183X.2004.00106.x<br>https://doi.org/10.3389/fnbeh.2014.00257<br>https://doi.org/10.1101/2020.04.28.066704<br>10.1016/s0278-5846(99)00081-0
	Food type and dispenser and if there were any restrictions on diet	10.1002/brb3.708<br>10.1016/j.physbeh.2005.06.013<br>10.1152/physiolgenomics.00018.2020<br>10.1016/j.physbeh.2005.07.008<br>10.1016/j.brainres.2012.06.004<br>10.3390/ani6010004<br>10.1007/s11011-017-0140-z<br>10.1016/j.neulet.2018.01.025
	Food type of dams before conception, during gestation or lactation	10.1016/j.neuroscience.2013.02.044<br>10.1016/j.psyneuen.2015.10.020<br>10.1016/j.neuint.2010.04.009<br>10.1016/j.bbr.2008.03.021<br>10.1080/1028415X.2017.1354958
	Cleaning frequency, bedding transfer	10.1258/la.2009.0080098
	IVC/isolator or open cages	10.1016/j.physbeh.2013.10.019<br>10.1111/gbb.12564<br>10.3390/ani10040746
**Generic- *in vivo* - Handling**	Sex of Experimenter	10.1038/nmeth.2935
	Lab policy on use of perfume/skin care	

	Researcher experienced/not	10.1111/j.1601-183X.2005.00121.x<br>10.1016/j.bbr.2014.06.017
	Stress history of the animal (early life/chronic/acute etc.)	10.1097/WNR.0000000000000243<br>10.1016/0149-7634(81)90005-1<br>10.1016/j.neuroscience.2005.06.068
	Habituation to handling	https://doi.org/10.1016/S0091-3057(97)00502-9<br>https://doi.org/10.1016/S0091-3057(02)00789-X<br>https://doi.org/10.1038/s41598-020-60530-4
	Handling method	10.1037/com0000147
**Test conditions**		
**Test room**	Temperature	10.1016/j.jtherbio.2019.102458<br>https://doi.org/10.1016/j.applanim.2016.08.005
	Humidity	10.3791/51785
	Background noise level	10.1177/0023677217711966<br>https://doi.org/10.1111/j.2044-8295.1971.tb02034.x<br>10.1016/0376-6357(94)90011-6
	Illumination Level	https://doi.org/10.1016/j.applanim.2016.08.005<br>https://link.springer.com/article/10.1007%2FBF00572269<br>10.1016/j.brainresbull.2006.12.009<br>https://doi.org/10.1016/0031-9384(94)00317-3
	Odour	10.1016/S0091-6773(72)80214-1
	Cleaning agents (type, concentration, and frequency of cleaning)	10.3791/51785
	Time of testing (morning vs afternoon)	10.1037/a0021200<br>https://doi.org/10.1038/s41598-019-44705-2<br>10.3389/fphar.2019.00237
	Frequency of testing	10.2466/pms.1998.86.3c.1179<br>10.1016/j.bbr.2011.11.042<br>10.2466/pms.1998.86.3c.1179<br>10.1111/j.2044-8295.1972.tb01312.x<br>10.1016/S0003-3472(73)80047-8
	Transportation from holding facility	10.1258/la.2009.0080098 ;
	Acclimatization to test room and duration of test	https://doi.org/10.1371/journal.pone.0048414
	Bedding material in the test box	10.1016/j.pbb.2010.05.013
**Administration of drugs**	Name and type of drug	10.1016/S0014-2999(03)01272-X<br>10.1016/s0278-5846(99)00081-0<br>10.1016/j.bbr.2014.06.017
	Formulation of drug solution	
	Drug Naïve	10.1007/s002130050738<br>10.1016/j.neulet.2007.12.020<br>10.1016/j.peptides.2009.02.002<br>10.1016/j.alcohol.2017.04.005<br>10.1016/j.euroneuro.2015.10.002<br>10.2174/1570162x13666150121105221
	Administration route	10.1055/s-2006-941557

	Volume (ml/kg)	
	Vehicle and vehicle volume	
	Frequency of dosing and dosing regimen	10.1017/S1461145711001283<br>10.1016/s0024-3205(03)00612-x
**Recording & Scoring**	Video Angle	
	Time of Recording (similar to time of testing)	https://doi.org/10.1038/s41598-019-44705-2<br>10.3389/fphar.2019.00237<br>10.1037/a0021200
	Duration of recording	
**Data analysis**	Software acquisition and analysis settings	10.1016/j.jneumeth.2017.05.026
	Manual/Automatic	10.4137/JCNSD.S13194<br>10.1038/s41386-020-0776-y
	Analysis parameters	
**Statistical analysis**	Multiple raters	10.1038/s41386-020-0776-y
	Pre-defined statistical analysis	10.1016/s0278-5846(99)00081-0<br>10.3389/fnbeh.2017.00026
	Appropriate choice of statistical method	10.1016/s0278-5846(99)00081-0
	Definition of unit of analysis	
	Precision of effect size	
**Equipment**	Apparatus Construction (floor surface, color, size)	https://doi.org/10.1152/physiolgenomics.90207.2008<br>https://link.springer.com/article/10.1007%2FBF00572269
	Thermometer	
	Camera and computer type	10.3758/s13428-017-0904-8 ;<br>10.1038/s41386-020-0776-y<br>10.1016/j.jneumeth.2017.05.026
	Analysis Software	https://doi.org/10.1089/zeb.2018.1662
	Infrared fine tuning	10.1016/j.physbeh.2016.02.014

Table S2: List of factors for the Western Blotting protocol

Category	Factor
**Tissue harvest conditions**	Method utilised for collection of tissue (perfusion/CO2/decapitation)
	Method for preservation of tissue (flash frozen/fixation)
	Tissue fasted
**Sample Preparation**	Multiple freeze thaw cycles affecting degradation
	Method of tissue homogenisation
	Buffer utilised for tissue homogenisation and sample preparation
	Fractionation procedure

	Protease inhibitors
	Temperature
	Storage
**Sample quantification**	Buffer compatibility
	Protein quantification method
**Polyacrylamide gel**	Type of gel utilised
	Manufacturer
	% gel
	Age/ lot
**Membrane**	Membrane type
	Age/lot
	Manufacturer
**Sample loading**	Amount of protein loaded onto gels
**Transfer conditions**	Protein size
	Transfer buffer
	Transfer time
	Current/voltage
**Blockng solution**	Type of blocking solution
	Concentration
	Cross-reactivity
**Primary antibody**	Specificity
	Titer
	Affinity
	Incubation time
	Source animal
	Concentration
	Lot
	Temperature
**Secondary antibody**	HRP conjugate enzyme activation level and activity
	Source animal
	Concentration
	Temperature
	incubation time,
**Washing**	Buffer

	Frequency
	Volume
	Duration
**Normalisation**	Loading control utilised
	Housekeeping proteins
**Detection**	Detection method
	Substrate type, lot, sensitivity
	Age of substrate
	Film age
	Type of imaging instrument
	Exposure time
**Quantification**	Quantification (densitometry)
	Software used
	Background subtraction
	Signal saturation
**Test conditions**	Inter-operator variability
